# Supplementary figures and images for: Somatic LINE-1 retrotransposition in cortical neurons and non-brain tissues of Rett patients and healthy individuals
Source: PLoS Genet. 2019 Apr 11;15(4):e1008043. doi: 10.1371/journal.pgen.1008043 (PMC6478352; doi:10.1371/journal.pgen.1008043)

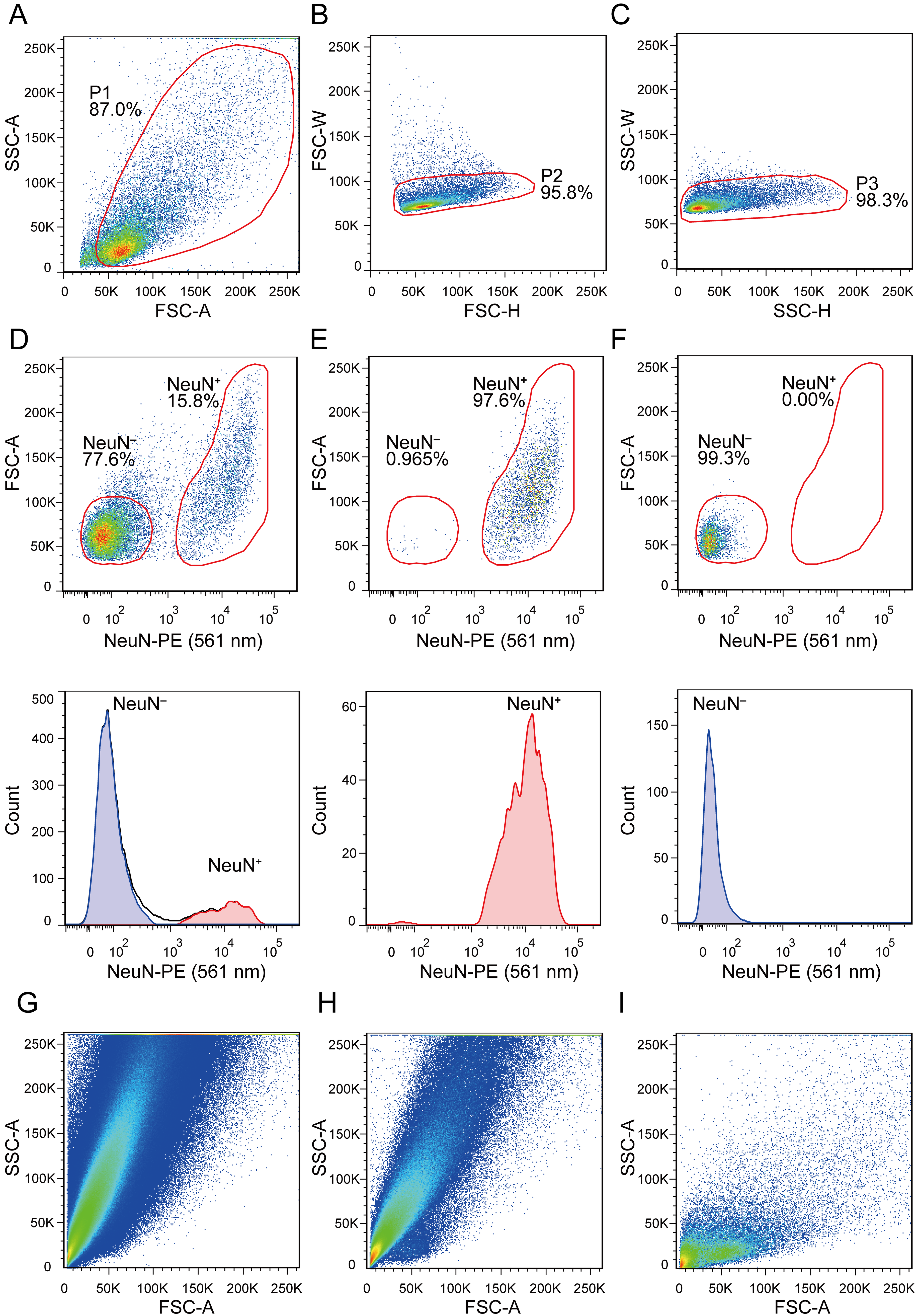

Supplement: S1 Fig — (A)–(D) Purify neuronal nuclei from human PFC. (A) The first gate (P1) was set as an FSC-A vs. SSC-A plot to discriminate the population containing small-size debris. (B)–(C) The second (P2) and third (P3) gates were set as FSC-H vs. FSC-W and SSC-H vs. SSC-W plots, respectively, to remove doublets and clumps. (D) Top: NeuN− and NeuN+ gates were set in the NeuN-PE (561 nm) vs. FSC-A plot. Bottom: a count plot of NeuN-stained nuclei. (E) Purity analysis of sorted neurons. Top: sorted NeuN+ nuclei were re-analyzed by FACS to confirm the sort purity. Bottom: a count plot of re-analyzed NeuN+ nuclei. (F) Purity analysis of sorted glia. Top: sorted NeuN− nuclei were re-analyzed by FACS to confirm the sort purity. Bottom: a count plot of re-analyzed NeuN− nuclei. (G) FSC vs. SSC plot of brain homogenate. Brain homogenate contained a huge amount of cell debris and myelin debris. (H) FSC vs. SSC plot of debris-detached single-nuclei homogenate. Minced brain tissue was soaked overnight before homogenization and then incubated with nonionic detergent, Nonidet P-40, to remove cell debris from nuclear membrane. (I) FSC vs. SSC plot of debris removed nuclei fraction. Cell debris and myelin were separated from nuclei using Percoll density gradient centrifugation. NeuN-PE, PE-conjugated anti-NeuN antibody. (TIF) [file pgen.1008043.s001.tif]

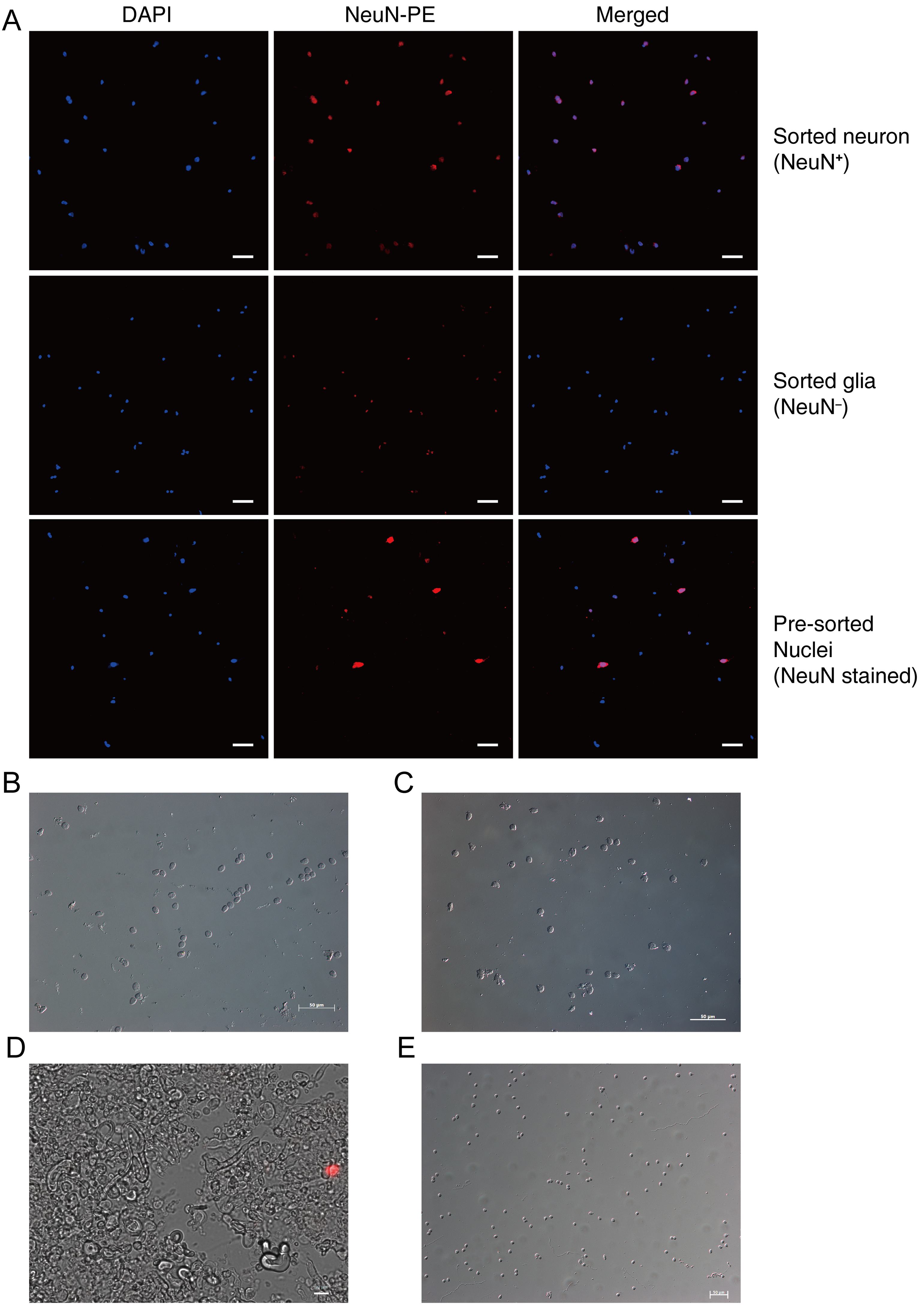

Supplement: S2 Fig — (A) Example of fluorescence microscopy confirmation of isolated nuclei. The purity of each fraction was > 95% for NeuN+ and NeuN− nuclei. Bar = 50 μm. (B)–(C) Examples of integrity confirmation using differential interference contrast (DIC) of sorted neurons (B) and glia (C). Bar = 50 μm. (D) Example of the myelin, lipid, and cell debris layers (12% Percoll) after Percoll density gradient centrifugation. Nuclei were stained with a red fluorescent nuclear counterstain, propidium iodide (PI). Bar = 20 μm. (E) Example of the nuclei fraction layer (35% Percoll) after Percoll density gradient centrifugation. Bar = 50 μm. DAPI, 4’,6-diamidino-2-phenylindole; NeuN-PE, PE-conjugated anti-NeuN antibody. (TIF) [file pgen.1008043.s002.tif]

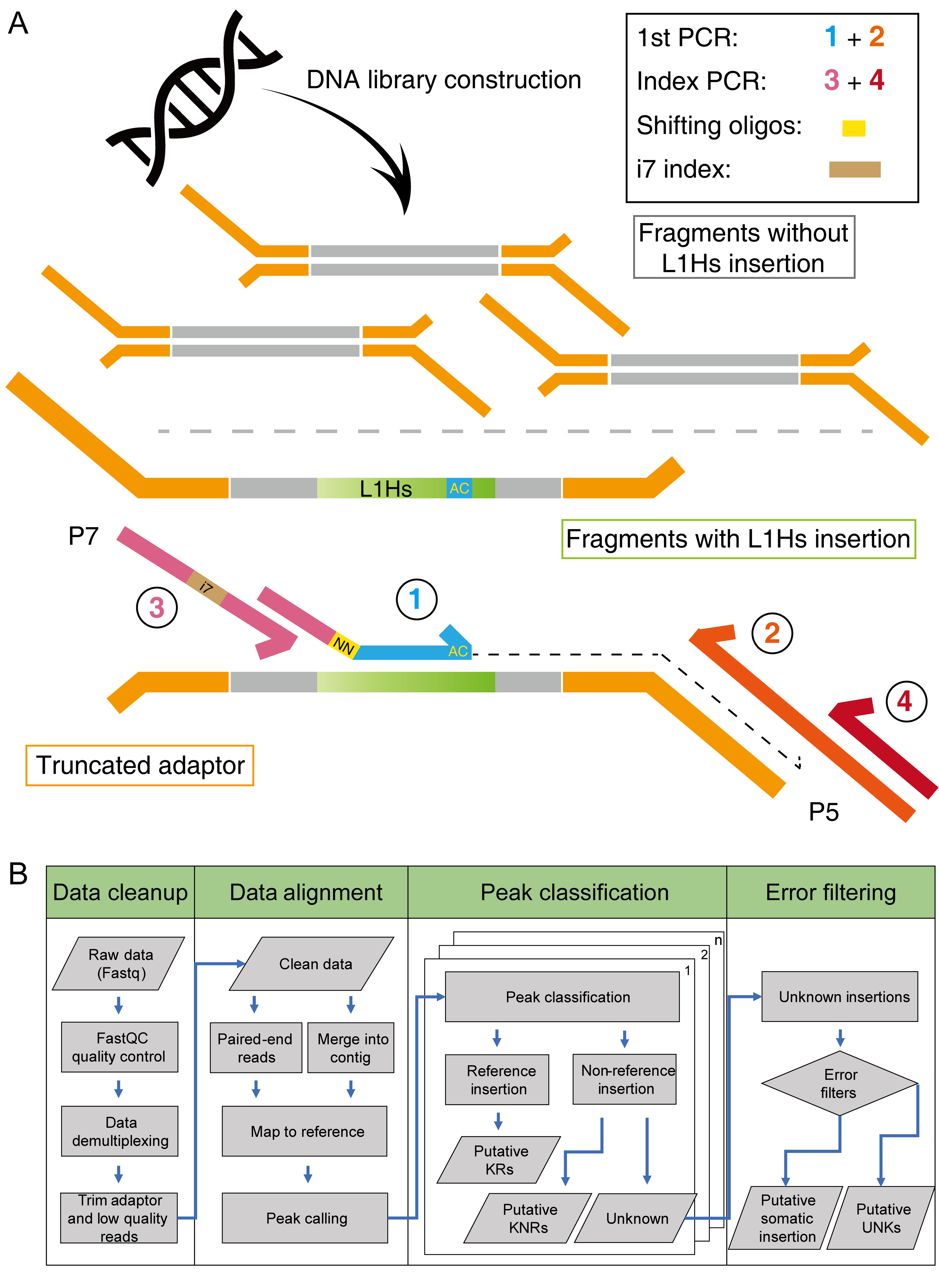

Supplement: S3 Fig — (A) Schematic of the HAT-seq library construction. The fragmented genomic DNA was ligated with P7 truncated adaptors, and then used as template for L1Hs amplification PCR. Primers 1 (P7_Ns_L1Hs) was specific to L1Hs diagnostic ‘‘AC” motif. See S1 Table for primer sequences. (B) Schematic of the HAT-seq data analysis pipeline; full details are provided in the Methods. (TIF) [file pgen.1008043.s003.tif]

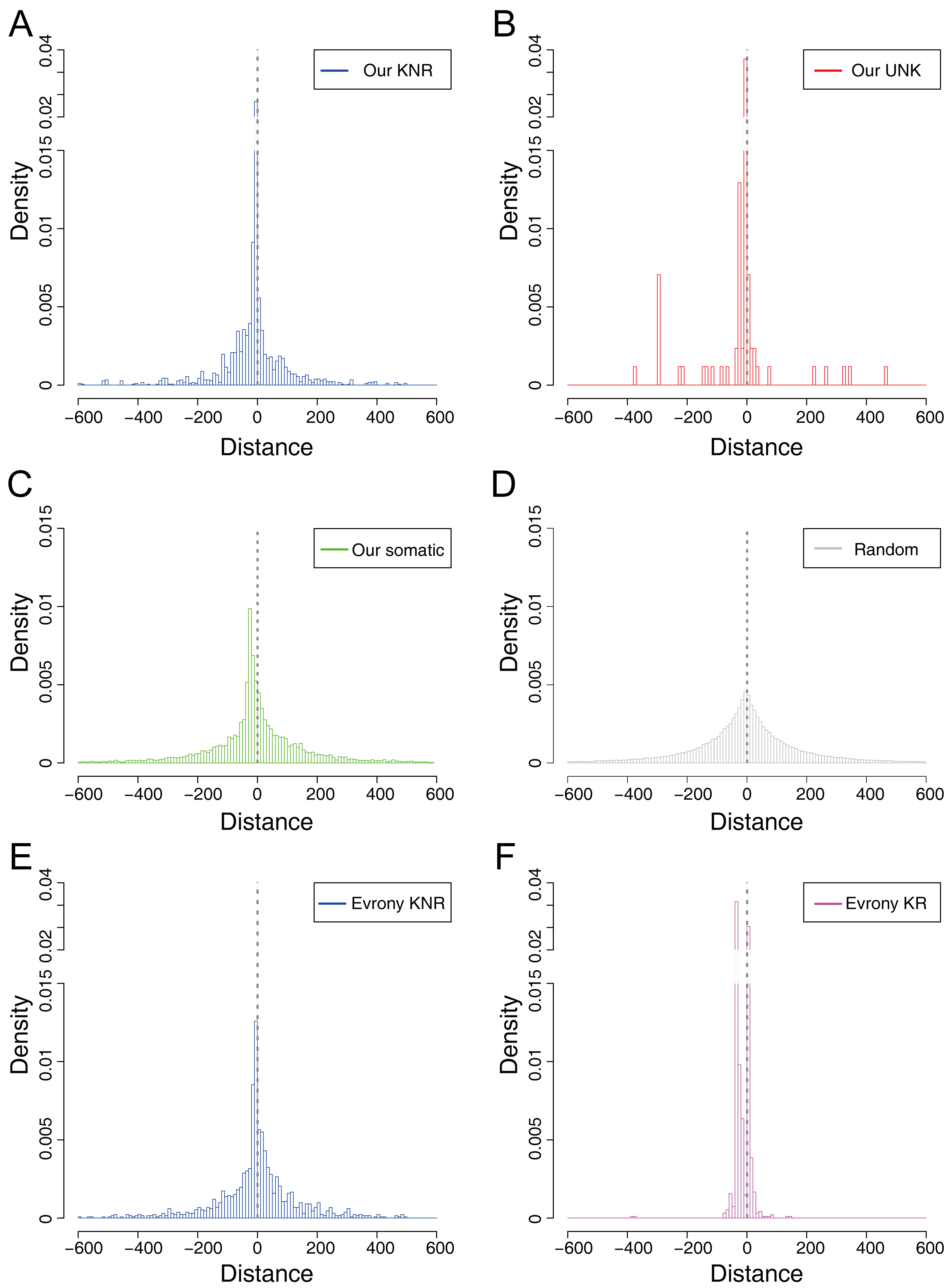

Supplement: S4 Fig — The density distributions of L1 EN motifs around germline KNR (A), UNK (B), somatic insertions (C), randomly sampled positions (D), “Evrony KR” (E), and “Evrony KNR” (F). The lists of “Evrony KR” and “Evrony KNR” were extracted from Evrony et al. 2012. The bin size of histogram was 10bp. L1 EN motifs included seven specific motifs (TTAAAA, TTAAGA, TTAGAA, TTGAAA, TTAAAG, CTAAAA, TCAAAA). (TIF) [file pgen.1008043.s004.tif]

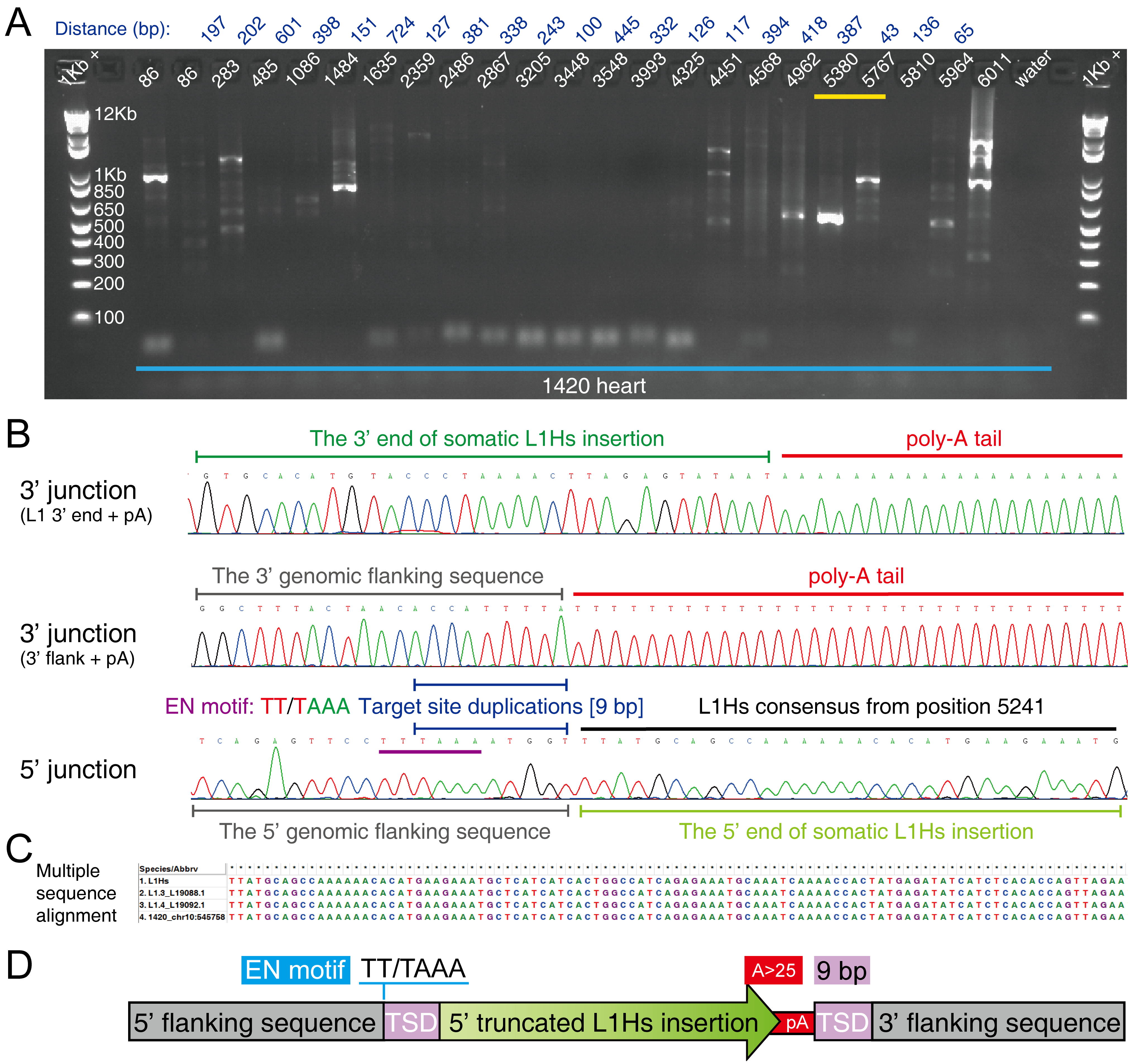

Supplement: S5 Fig — (A) The agarose gel image of 5’ junction nested PCR validation for the heart-specific L1Hs insertion in the Rett patient (UMB#1420). The locations of primers used in 5’ junction PCR assays were labeled on the top of each lane, where primers with the prime symbol denoted semi-nested PCR assays. The distances between each two adjacent 5’ step-wise primers were labeled on the top (dark blue). The yellow line highlighted the expected stair-step bands in 5’ junction PCR. 1Kb +: 1 Kb Plus DNA ladder. (B) The Sanger sequencing chromatograms of the 3’ and 5’ junctions of the somatic insertion (1420_chr10:545758). The L1 EN motif and TSD were indicated by purple and blue lines. (C) Multiple sequence alignment of the 5’ end between the identified somatic insertion and three L1Hs consensus sequences (L1Hs Repbase consensus and two hot L1s in human [L1.3 and L1.4]). (D) The schematic structure of the highly 5’ truncated (~800 bp) L1Hs insertion 1420_chr10:545758. (TIF) [file pgen.1008043.s005.tif]

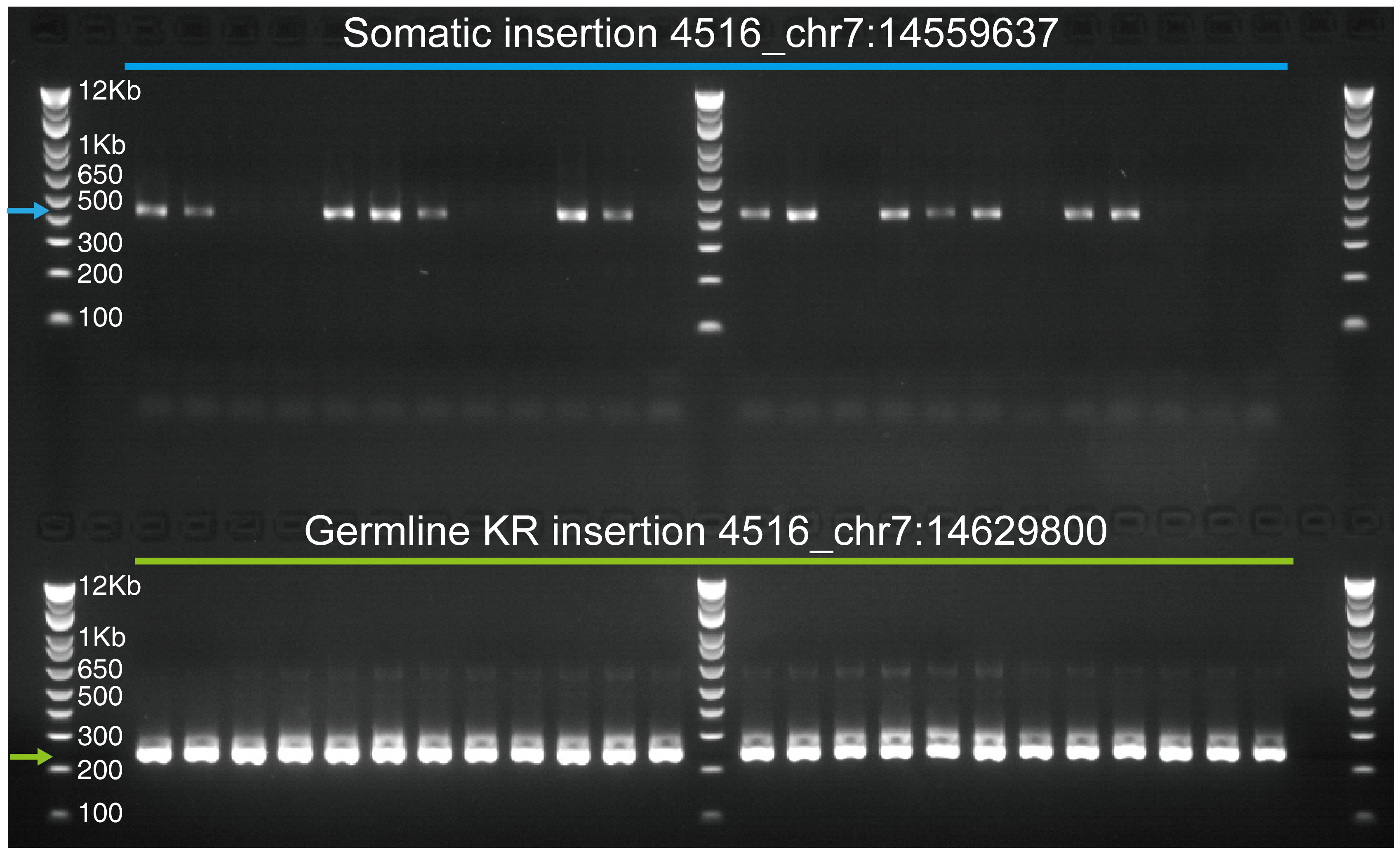

Supplement: S6 Fig — The somatic insertion 4516_chr7:14559637 was present in 14 out of 24 nested 3’ PCR wells, compared to 24 out of 24 wells for a germline KR insertion (chr7:14629800) from the same donor. DNA sample was diluted to ~300 cells per well. Blue and green arrows indicated bands with target size. (TIF) [file pgen.1008043.s006.tif]

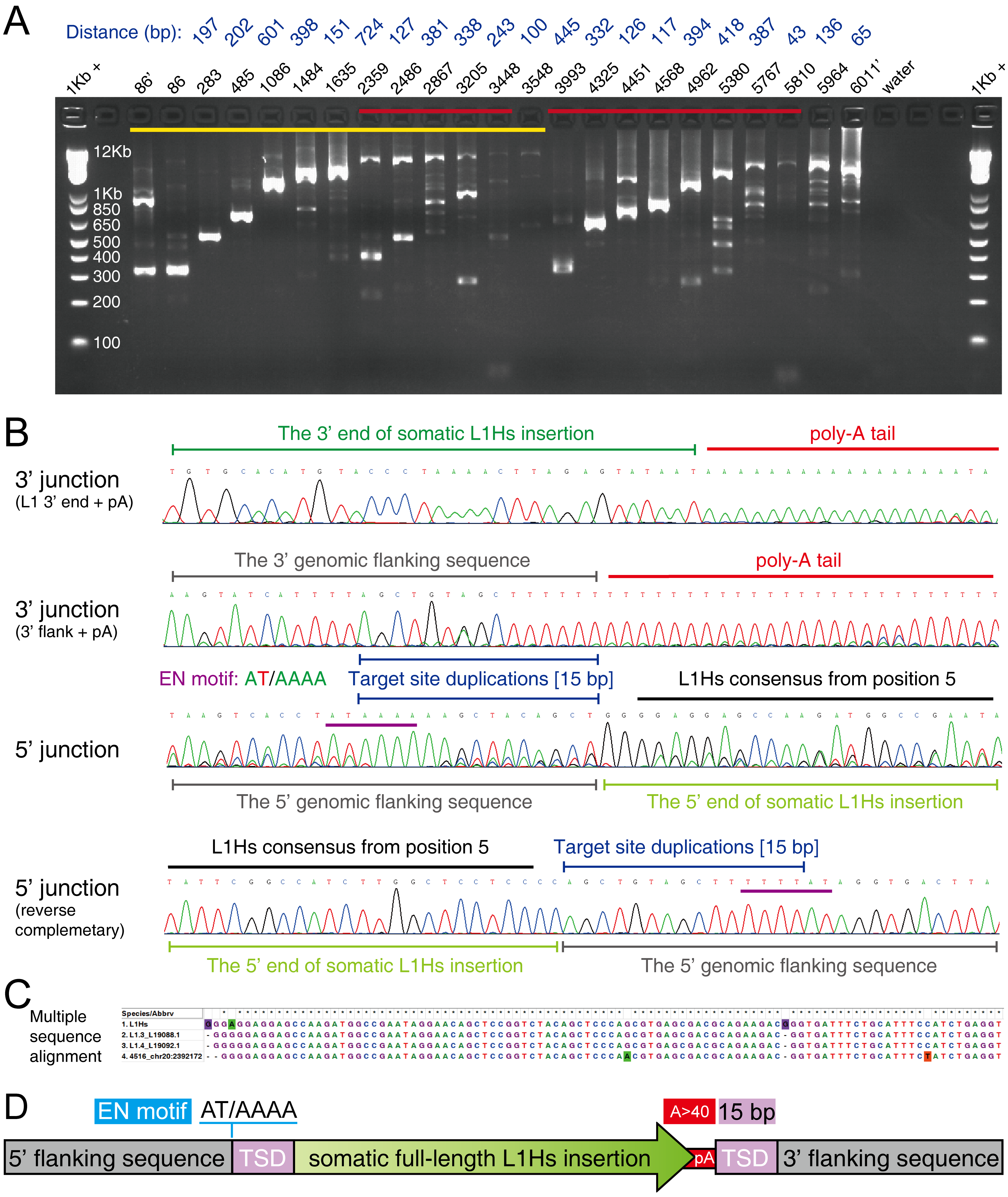

Supplement: S7 Fig — (A) The agarose gel image of 5’ junction nested PCR validation for the embryonic somatic L1Hs insertion (4516_chr20:2392172) in the Rett patient (UMB#4516). The locations of primers used in 5’ junction PCR assays were labeled on the top of each lane. Step-wise primers with the prime symbol were used twice in semi-nested PCR assays. The distances between each primer pairs were labeled on the top (dark blue). The yellow line highlighted the expected stair-step bands in 5’ junction PCR, while the red lines indicated false positives resulted from non-specific amplification of L1PA subfamilies. 1Kb +: 1 Kb Plus DNA ladder. (B) The Sanger sequencing chromatograms of the 3’ and 5’ junctions of somatic insertion (4516_chr20:2392172). The nucleotides shifted chromatogram in 5’ junction might result from the DNA polymerase slippage at homopolymers in the upstream region (L1MB3 element), and its sequence was confirmed from the reverse direction. The L1 EN motif and TSD were indicated by purple and blue lines. (C) Multiple sequence alignment of the 5’ end between the identified somatic insertion and three L1Hs consensus sequences (L1Hs Repbase consensus and two hot L1s in human [L1.3 and L1.4]). (D) The schematic structure of 4516_chr20:2392172. (TIF) [file pgen.1008043.s007.tif]

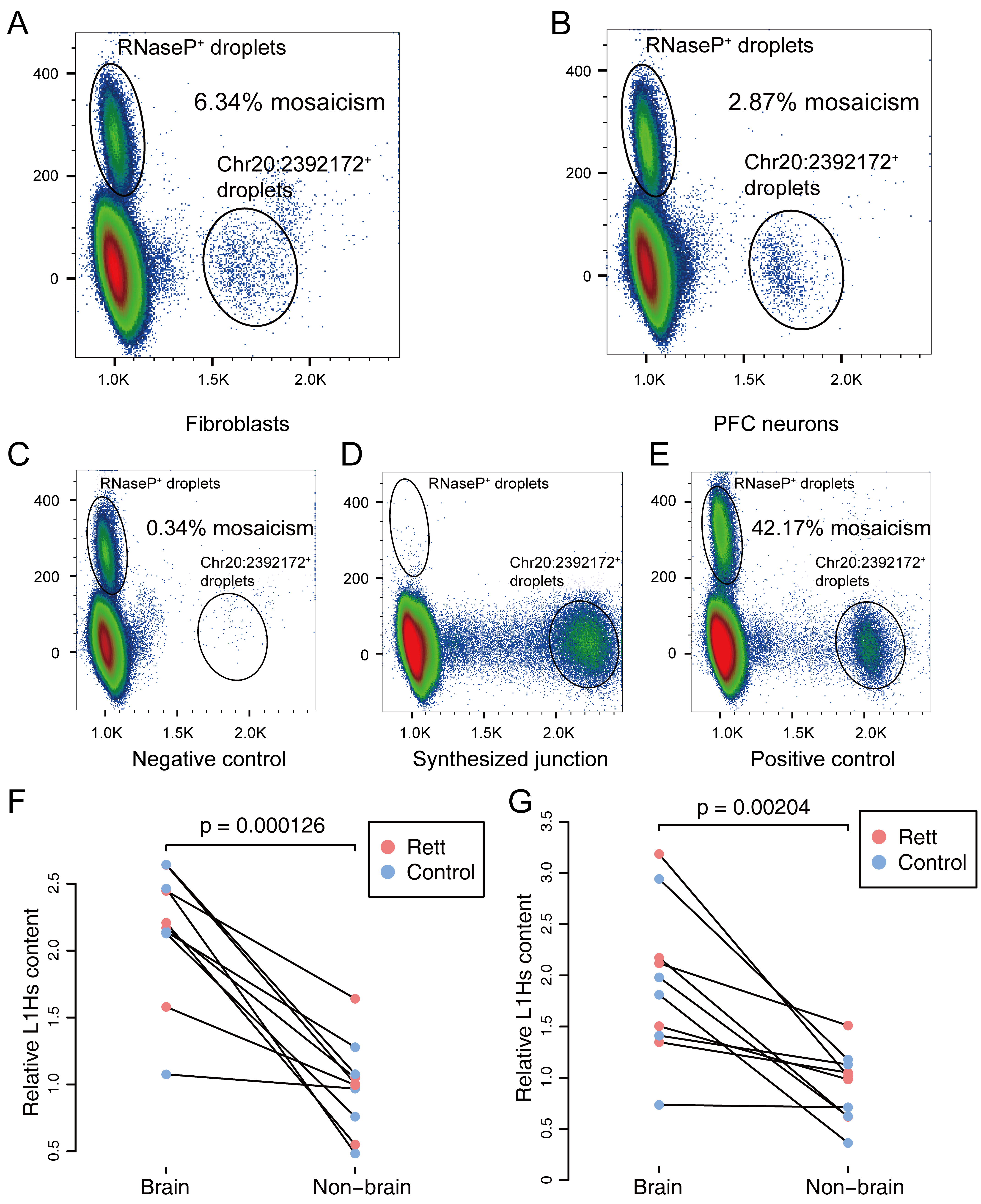

Supplement: S8 Fig — (A)–(E) Droplet digital PCR (ddPCR) assays to quantify mosaicism (percentage of cells) of somatic L1Hs insertions at chr20:2392172 in fibroblasts (A) and PFC neurons (B) from Rett patient UMB#4516. Fragmented ACC1 blood gDNA was used as template for negative control assay (C). A mixed template containing fragmented ACC1 blood gDNA and diluted synthesized L1Hs genome junction oligos (D) was used for positive control assay (E). RNaseP served as a genomic copy number reference (copy number = 2). L1Hs and RNaseP assays were labeled with FAM and VIC, respectively. (F)–(G) Relative somatic L1Hs content in PFC neurons and non-brain tissue from the same donor, normalized by the read count of KRs (F) or UNKs (G) from the same tissue sample. (TIF) [file pgen.1008043.s008.tif]
