# Supplementary material for: Somatic LINE-1 retrotransposition in cortical neurons and non-brain tissues of Rett patients and healthy individuals
Source: PLoS Genet. 2019 Apr 11;15(4):e1008043. doi: 10.1371/journal.pgen.1008043 (PMC6478352; doi:10.1371/journal.pgen.1008043)
Supplement: S2 Appendix — (PDF) [file pgen.1008043.s026.pdf]

Appendix 2. Identification of ACC1-specific insertions and their zygosity

To identify ACC1-specific insertions, at the first step, we identified 172 ACC1 non-reference germline L1Hs insertions using HAT-seq. Next, 64 of them were confirmed to be ACC1-specific by 3' junction PCR (3' PCR) analysis of gDNA from ACC1 and ACC2. At last, we performed full-length PCR to determine the zygosity of ACC1-specific insertions.

<u>Identification of ACC1-specific insertions</u>

Validation primer sequences used for each candidate insertion and the predicted target amplicon sizes of 3' junction PCR products could be found in S2 Table. The IDs of ACC1-specific insertions were labeled at the top of agarose gel image. A: ACC1 gDNA; Z: ACC2 gDNA; M: 100 bp Plus DNA ladder; NTC: water. For details, see details in Methods "Positive control experiments" and "L1 3' PCR and full-length PCR validation".

<u>Zygosity of ACC1-specific insertions</u>

Validation primer sequences used for each ACC1-specific insertion and the predicted empty site sizes of full-length PCR products could be found in S2 Table. The IDs of ACC1-specific insertions were labeled at the top of agarose gel image. M: 100 bp Plus DNA ladder; NTC: water. For details, see Methods "L1 3' PCR and full-length PCR validation". Several sites were tested by full-length PCR for multiple times to robustly determine their zygosity.

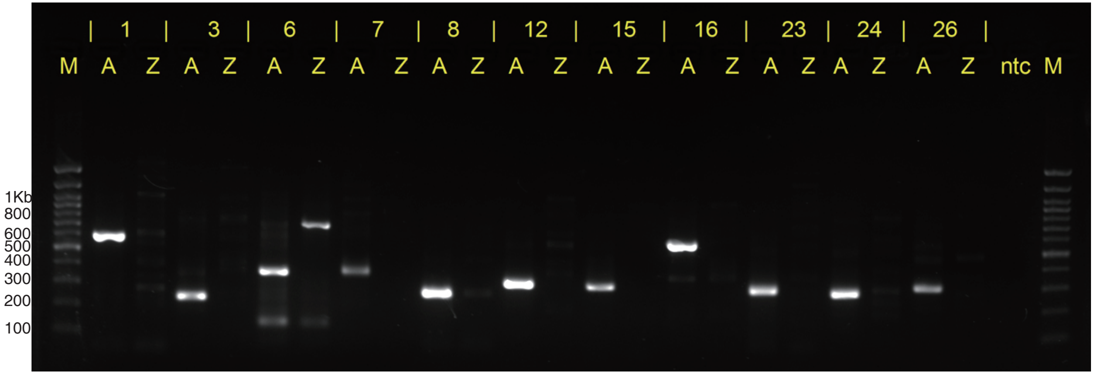

Identification of ACC1-specific insertions agarose gel image 1

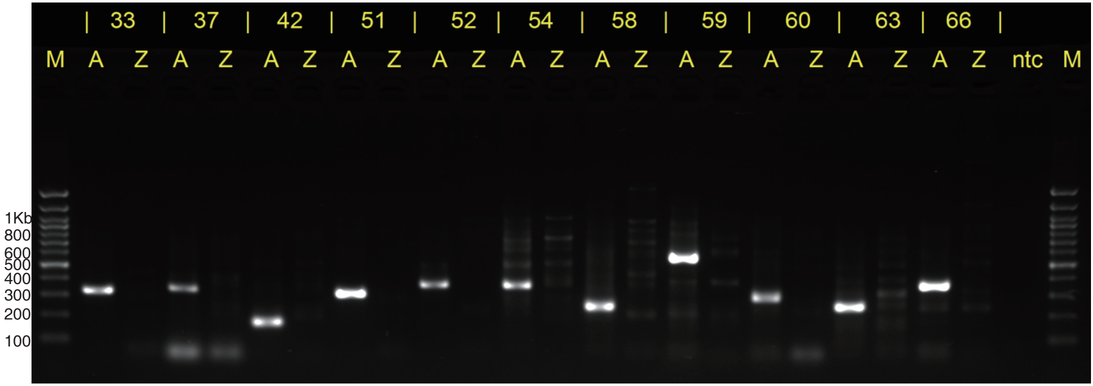

Identification of ACC1-specific insertions agarose gel image 2

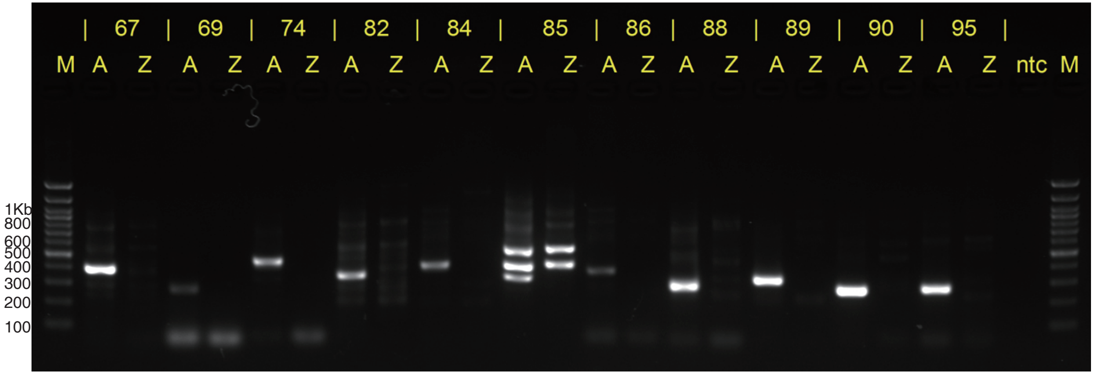

Identification of ACC1-specific insertions agarose gel image 3

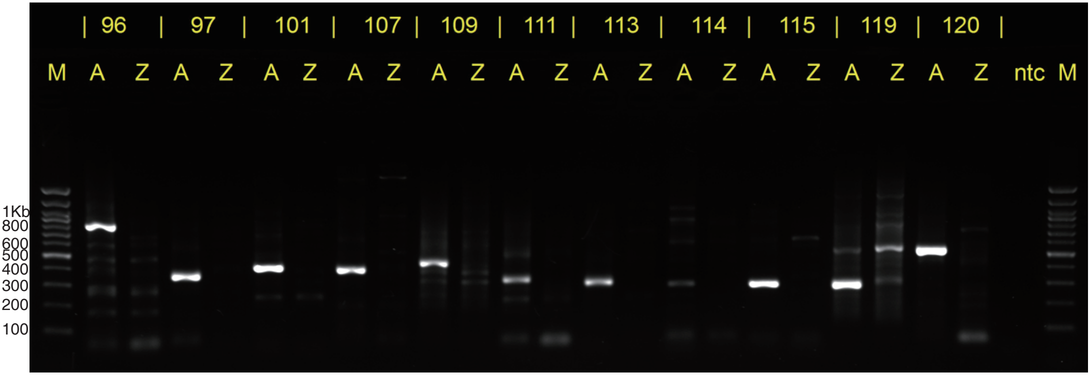

Identification of ACC1-specific insertions agarose gel image 4

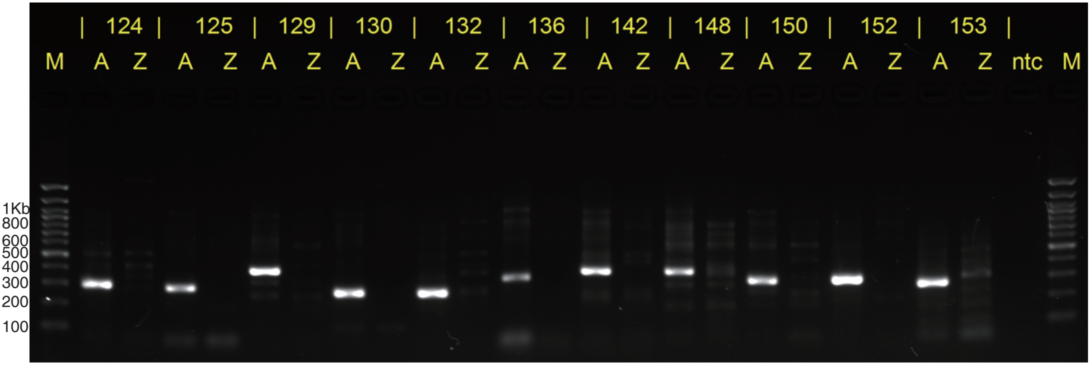

Identification of ACC1-specific insertions agarose gel image 5

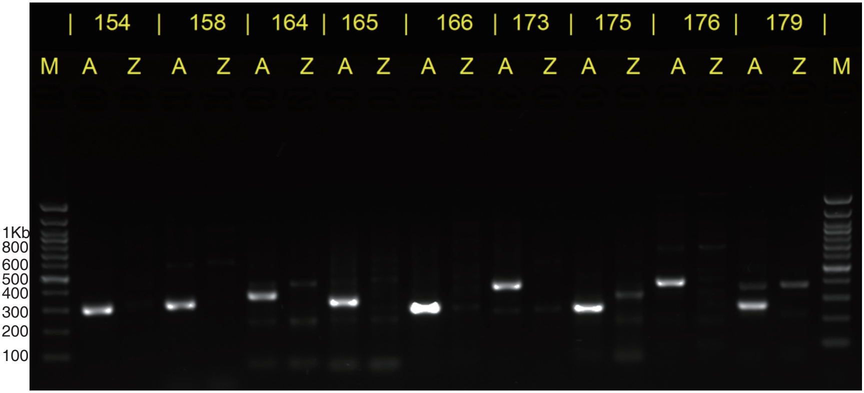

Identification of ACC1-specific insertions agarose gel image 6

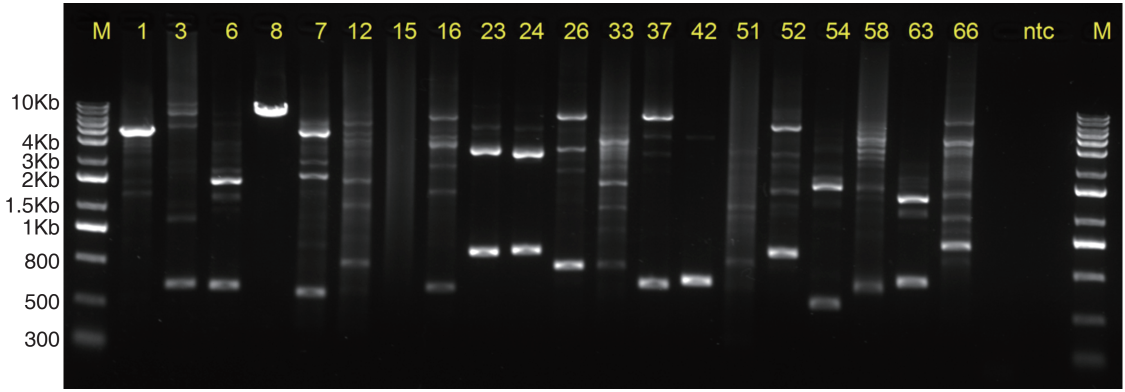

Zygosity of ACC1-specific insertions agarose gel image 1

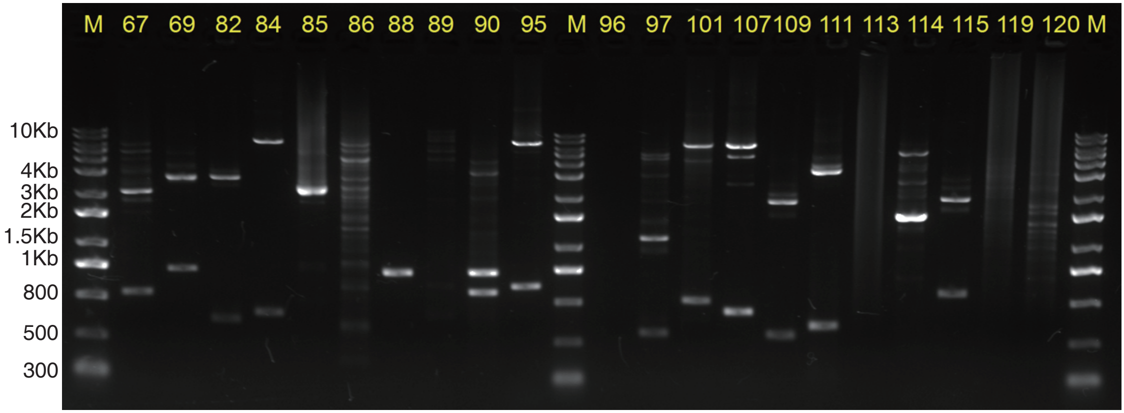

Zygosity of ACC1-specific insertions agarose gel image 2

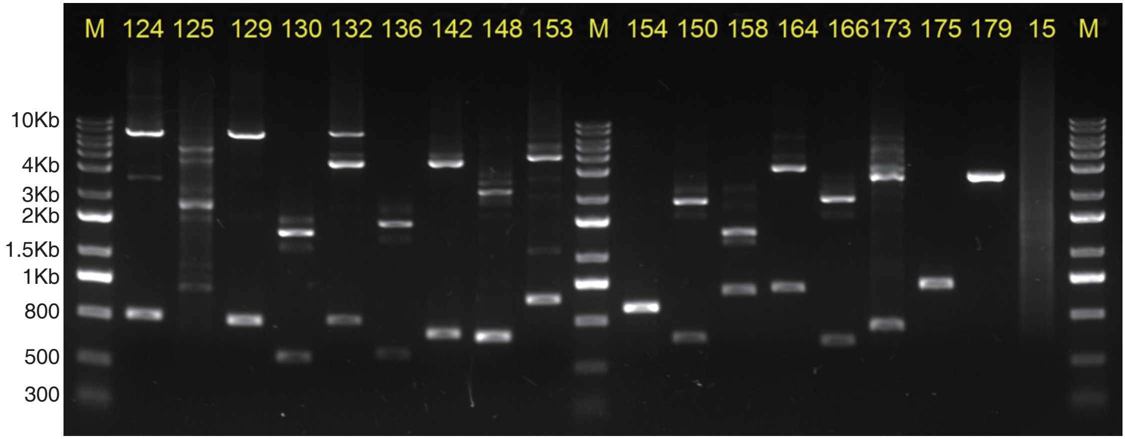

Zygosity of ACC1-specific insertions agarose gel image 3

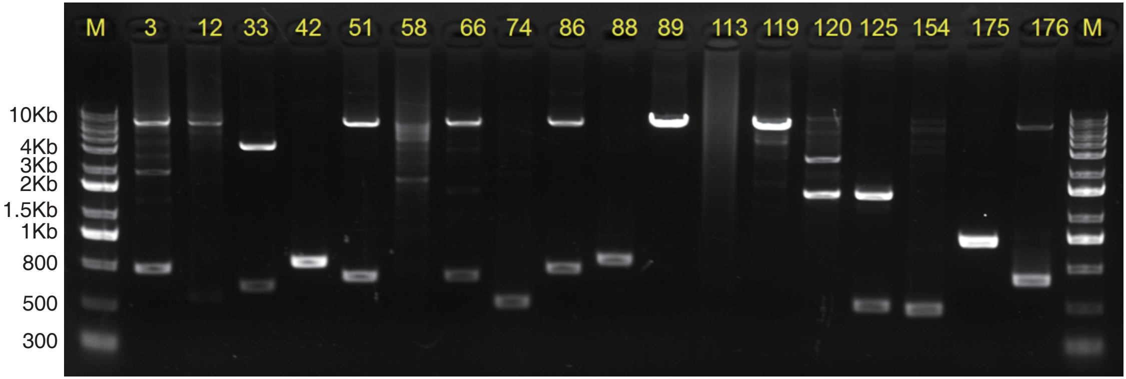

Zygosity of ACC1-specific insertions agarose gel image 4

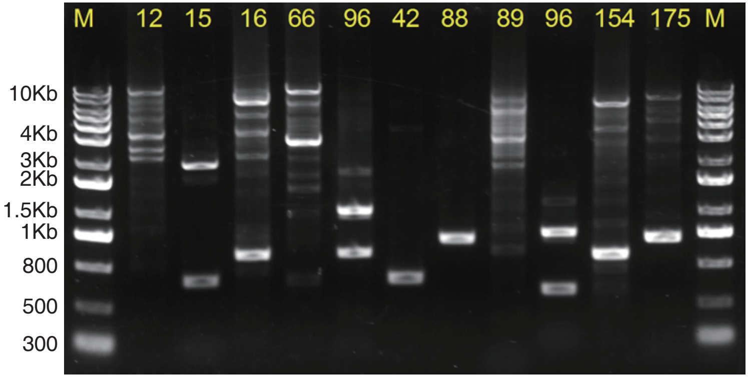

Zygosity of ACC1-specific insertions agarose gel image 5

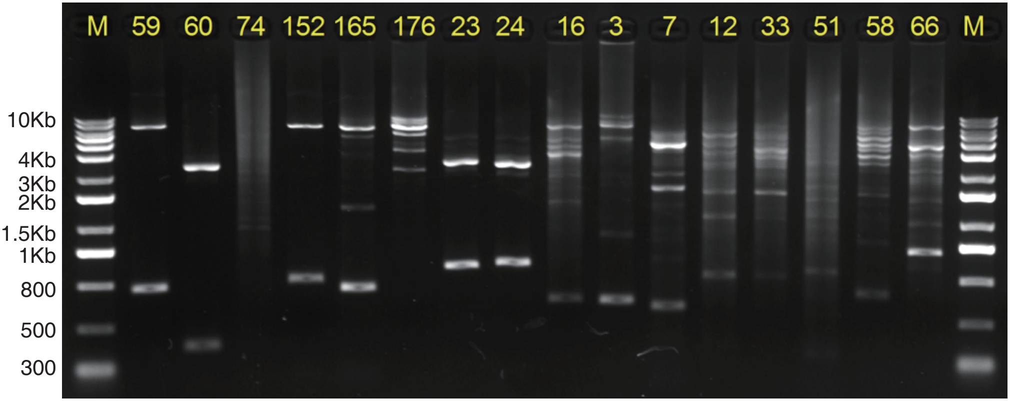

Zygosity of ACC1-specific insertions agarose gel image 6
