# Supplementary material for: Somatic LINE-1 retrotransposition in cortical neurons and non-brain tissues of Rett patients and healthy individuals
Source: PLoS Genet. 2019 Apr 11;15(4):e1008043. doi: 10.1371/journal.pgen.1008043 (PMC6478352; doi:10.1371/journal.pgen.1008043)

Appendix 5. Experimental validation of polymorphic germline L1Hs insertions.

To experimentally validate the HAT-seq predicted germline insertions, we performed 3' junction PCR validation on a random subset of polymorphic insertions from among the ten individuals, including 8 sites out of 160 polymorphic KRs, 20 sites out of 451 KNRs, and 2 sites out of 48 UNKs (S8 Table [3' PCR NOTE]).

<u>3' PCR validation of polymorphic germline L1Hs insertions</u>

Validation primer sequences used for each candidate insertion, the predicted target amplicon sizes of 3' junction PCR products, and statistics on 3' PCR validation results could be found in S16 Table. The IDs of ten individuals were labeled at the top of each page and agarose gel image. M: 100 bp Plus DNA ladder. For details, see sections in the Materials and Methods "L1 3' PCR and full-length PCR validation".

Germline L1Hs KR insertions validation using 3' junction PCR

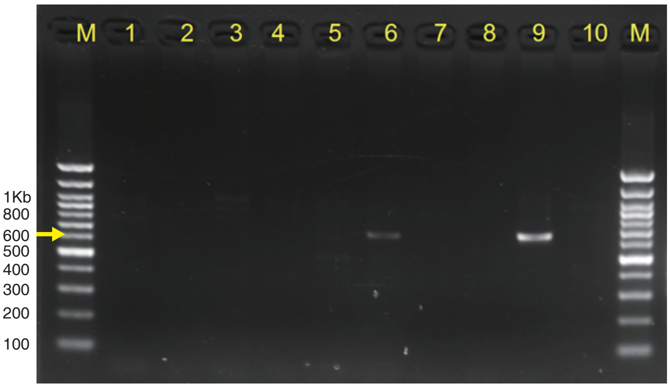

KR_1

M	1	2	3	4	5	6	7	8	9	10	M
100 bp +	1815	1571	4516	1846	4852	1347	4882	4591	1420	1455	

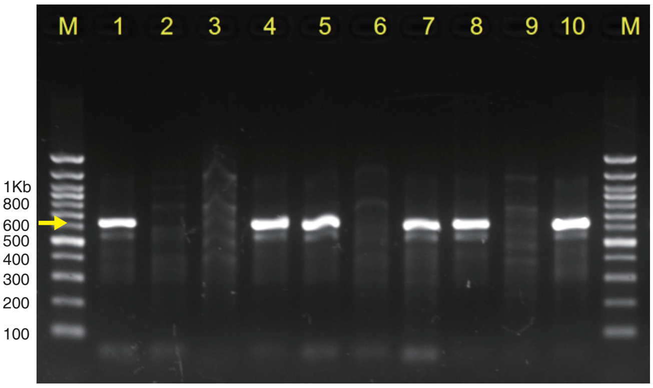

KR_3

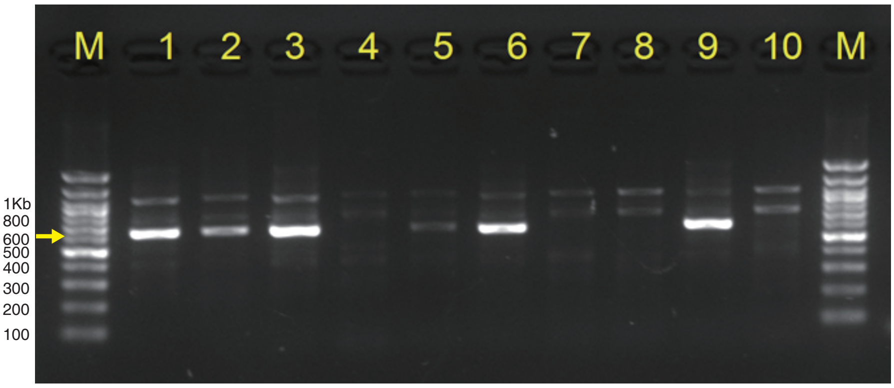

KR_4

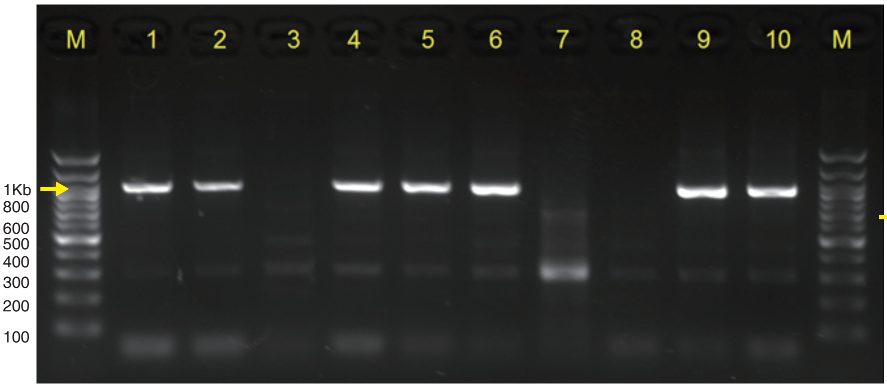

KR_5

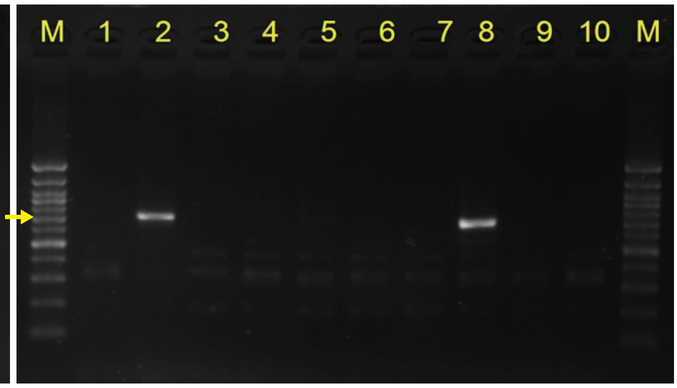

KR_6

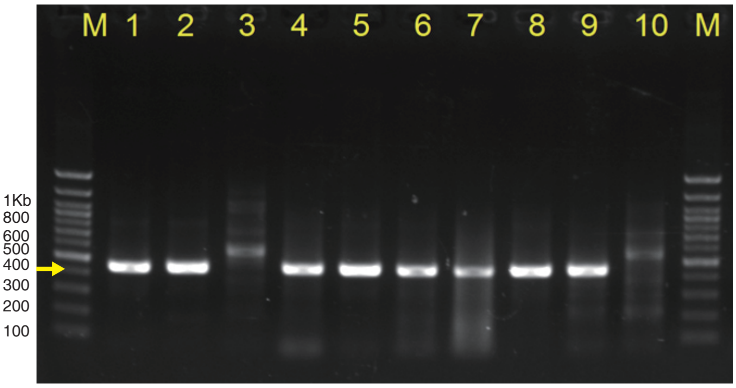

KR_7

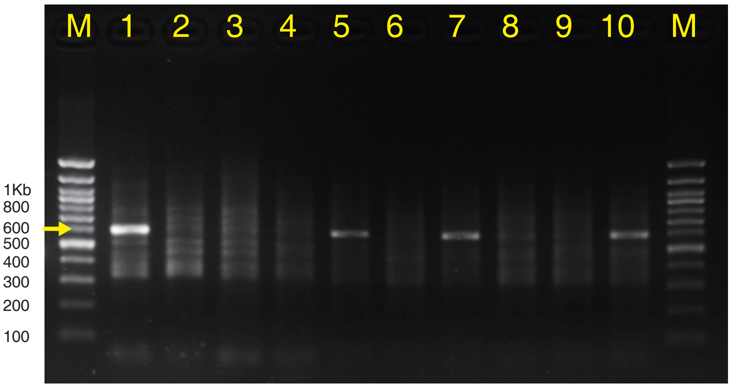

KR_8

Germline L1Hs KNR insertions validation using 3' junction PCR

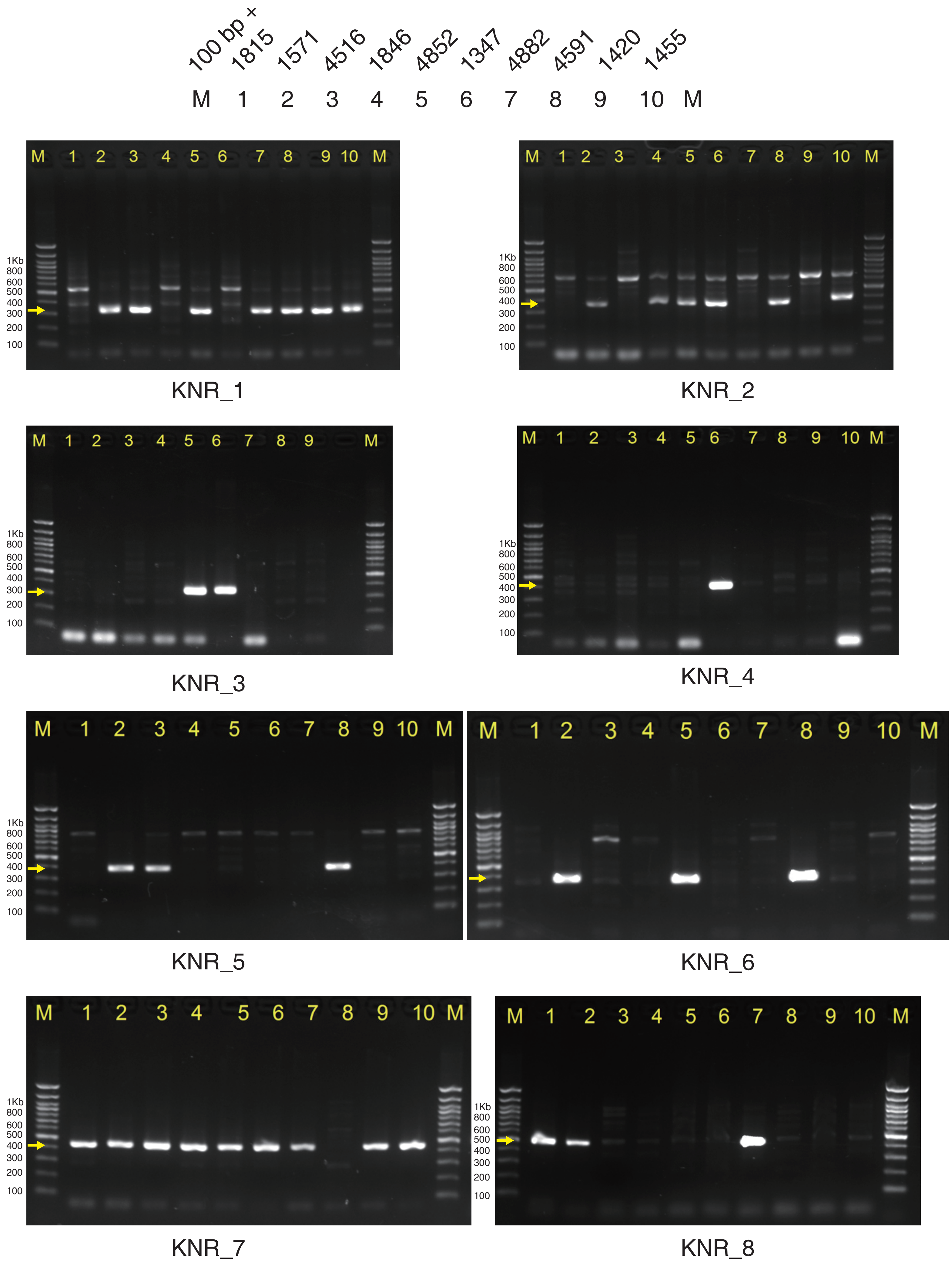

Germline L1Hs KNR insertions validation using 3' junction PCR

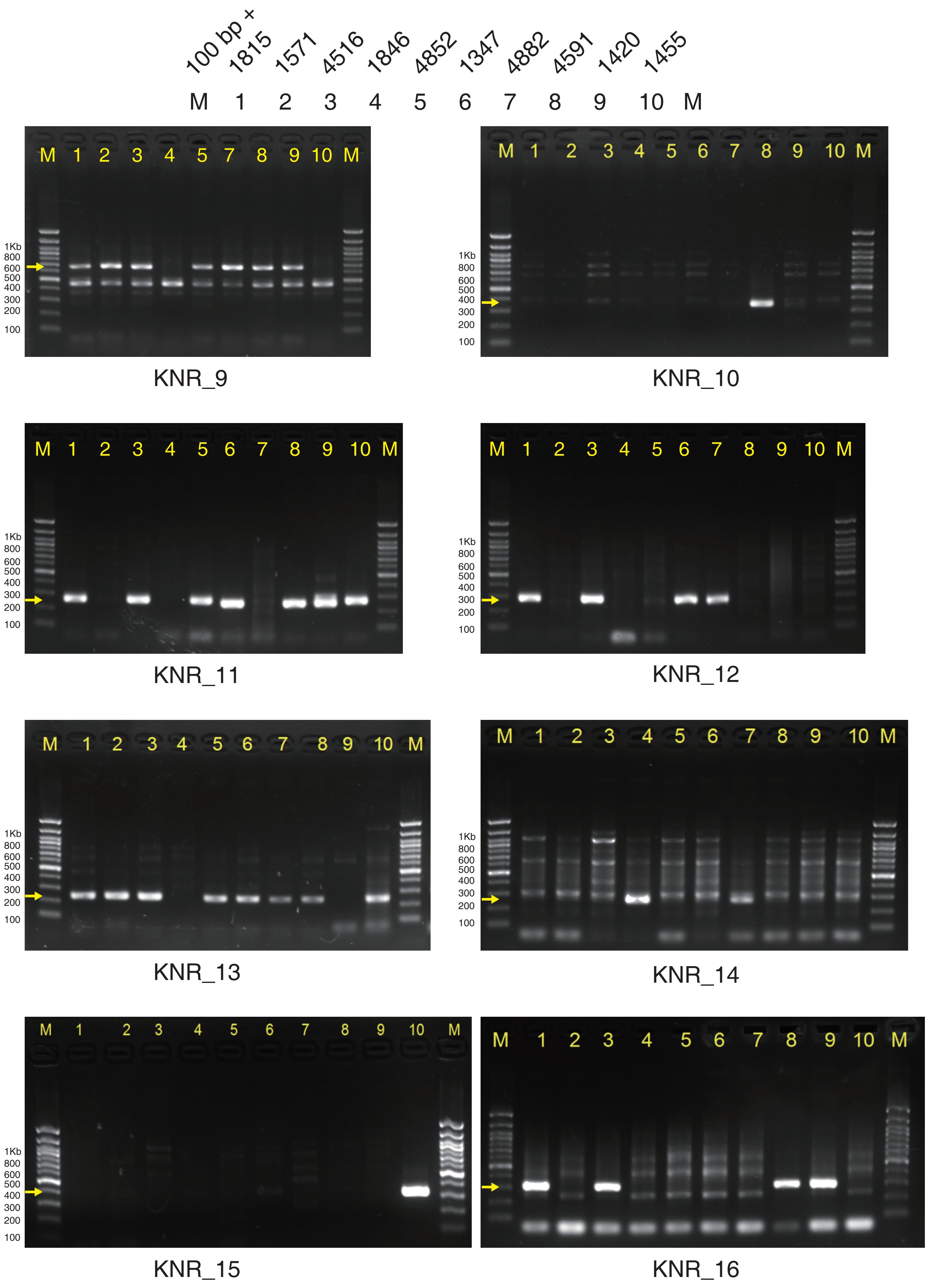

Germline L1Hs KNR insertions validation using 3’ junction PCR

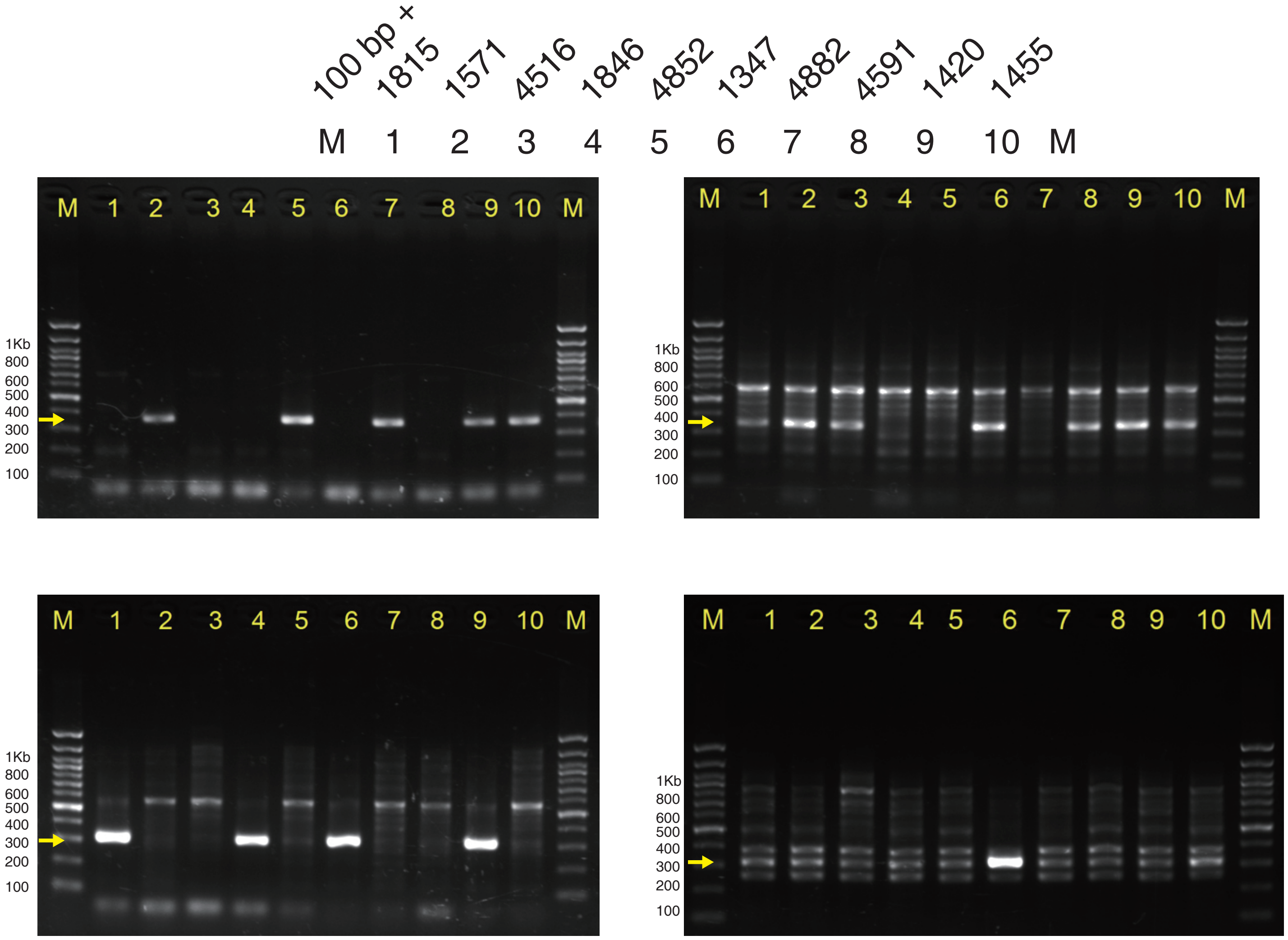

Germline L1Hs UNK insertions validation using 3’ junction PCR

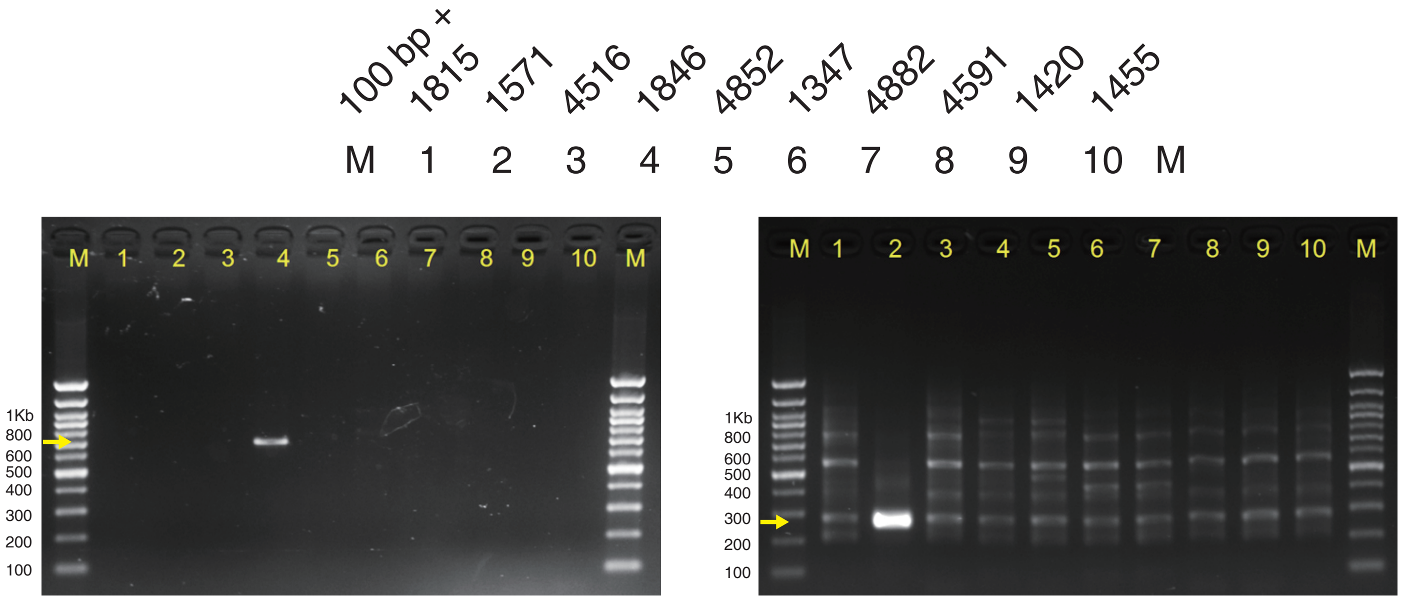

Supplement: S5 Appendix — (PDF) [file pgen.1008043.s029.pdf]
